# Cognitive Processes Underlying Reading Improvement during a Rhythm-Based Intervention. A Small-Scale Investigation of Italian Children with Dyslexia

**DOI:** 10.3390/children6080091

**Published:** 2019-08-08

**Authors:** Alice Cancer, Giulia Stievano, Gabriella Pace, Alessia Colombo, Alessandro Antonietti

**Affiliations:** Department of Psychology, Catholic University of the Sacred Heart, 20123 Milan, Italy

**Keywords:** reading, developmental dyslexia, rhythm, intervention, working memory, attention

## Abstract

Music and rhythm-based training programs to improve reading are a novel approach to treatment of developmental dyslexia and have attracted the attention of trainers and researchers. Experimental studies demonstrating poor basic auditory processing abilities in individuals with dyslexia suggest they should be effective. On this basis, the efficacy of a novel rhythm-based intervention, Rhythmic Reading Training (RRT), was recently investigated and found to improve reading skills in Italian children with dyslexia, but its mode of action remains somewhat unclear. In this study, 19 children and preadolescents with dyslexia received 20 sessions of RRT over 10 weeks. Gains in a set of reading-related cognitive abilities—verbal working memory, auditory, and visual attention, and rhythm processing—were measured, along with reading outcomes. Analysis of the specific contribution of cognitive subprocesses to the primary effect of RRT highlighted that reading speed improvement during the intervention was related to rhythm and auditory discrimination abilities as well as verbal working memory. The relationships among specific reading parameters and the neuropsychological profile of participants are discussed.

## 1. Introduction

Innovative interventions for dyslexia based on new scientific evidence and aetiological theories have recently been proposed. The aim of these interventions is to maximize reading improvement whilst optimizing the duration of the intervention and fostering participants’ motivation and engagement. These novel, process-based interventions are designed to target specific cognitive processes that are impaired in dyslexia, such as rapid automatized naming [1] and visuo-spatial abilities [2]. Temporal auditory processing has also been shown to have a central role in determining phonological and reading deficits in dyslexia (e.g., [3,4,5]). Children with dyslexia are less sensitive than developmentally typical children to the metrical structure of both verbal and nonverbal sounds [6], and rhythm discrimination ability was found to be a predictor of both reading and phonological abilities in both developmentally typical children and children with dyslexia [6,7]. An auditory processing impairment that specifically affects processing of the temporal component of sounds interferes with the development of phonological processing and phoneme–grapheme mapping, which are crucial for reading acquisition [8,9]. The relationship between rhythm processing and reading led researchers to test the hypothesis that musical training could improve dyslexia-related difficulties, with promising results [10,11,12,13].

On the basis of these findings, a rhythm-based computerized training program to improve the reading abilities of Italian individuals with reading problems was designed, Rhythmic Reading Training (RRT; [14]). Although RRT includes a rhythmic component, it consists of reading exercises designed to target specific reading subprocesses, namely, syllabic recognition (RRT’s Section 1, ‘Syllables’), phonological awareness (Section 2, ‘Merging’), grapheme-to-phoneme mapping, and lexical recognition (Section 3, ‘Words and Non-words’). The main characteristic of RRT exercises is the synchronization between reading and a metronomic, isochronous acoustic rhythm. Trainees have to adjust their reading to synchronize it to the acoustic rhythm. The tempo of the rhythm and the complexity of the verbal stimuli are increased gradually during each training session. Starting speeds can be matched to individual participants’ reading level by the supervisor of the training session. A visual cue to the verbal stimuli that is synchronized with rhythm can be added to support performance when necessary.

The efficacy of RRT has already been investigated in several test-training-retest studies (for a review: [15]) on primary and junior high school students with dyslexia. The program produced improvements in reading speed and accuracy which are higher than those coming from natural development [16]. The success of the intervention was not dependent on setting or the musical expertise of participants [15]. Moreover, the efficacy of the RRT was found to be comparable to that of other validated, multimodal approaches [15,17]. Comparative analyses showed that the RRT tended to produce greater effects on reading speed than on reading accuracy.

The goal of the small-scale investigation presented here was to investigate the mechanisms underlying the efficacy of RRT by measuring a set of cognitive abilities that were hypothesized to improve along with the primary outcome. The selection of reading-related subprocesses was based on previous research on predictors of reading in dyslexia. We assessed verbal short-term memory and working memory [18,19,20], auditory and visual attention [21,22], and rhythm and sound processing [6,7,23], along with reading outcomes, before and after the intervention. We decided to exclude phonological measures because they are poorly discriminative since readers of transparent languages tend to score highly in phonological awareness tests [24]. The specific aims of this study were to explore: (1) the relationship between pretraining measures of reading and reading-related abilities; (2) the effect of RRT on the primary outcome (reading ability) and the secondary outcomes; (3) the relationship between baseline neuropsychological profile and reading improvements during training; (4) the relationship between changes in reading skill and secondary outcomes during RRT; and (5) the relationship between reading outcome and the type of exercise delivered during training, in order to control for effects due to the type of exercises used to deliver the training.

## 2. Materials and Methods

### 2.1. Participants

Nineteen Italian students aged 8–14 years (M = 10.8; SD = 1.35; F = 63.2%) participated in the study. Participants had been diagnosed with dyslexia (ICD-10 code: F81.0) from professionals in three Neuropsychiatry Units in Milan, Italy, who followed the Italian standardized diagnostic procedure for specific learning disabilities (SLD) [25]. Students who had other neuropsychiatric or psychopathological conditions, such as attention deficit-hyperactivity disorder (ADHD), emotional dysregulation, autism spectrum disorder, or cognitive disability were excluded. Normal intelligence was a prerequisite for participation (full scale IQ ≥ 80; sample IQ: M = 99.4; SD = 11.1). Most participants (81.2%) had at least one comorbid SLD (i.e., dysorthography, dysgraphia, dyscalculia). Parents of eligible participants were contacted by the researchers to obtained written, informed consent. No compensation was given for participation. The study was conducted in accordance with the Declaration of Helsinki and approved by the Catholic University of the Sacred Heart (Milan, Italy) Research Ethics Committee.

### 2.2. Procedure

Participants received 20 individual sessions of RRT from psychologists specialized in RRT over a 10-week period. The psychologists who delivered the training underwent a specific programme of theoretical and practical training supervised by the authors of the intervention prior to their involvement in the study. All training sessions consisted of 30 min of computerized exercises and 30 min of musical games and activities [26] using small instruments (e.g., bongo drums, maracas, xylophone) that were designed to improve auditory processing abilities, which are often impaired in children with dyslexia (for a review: [27]). The two types of activity were alternated throughout sessions to promote motivation and engagement. All participants received the prescribed number of sessions and had not previously been exposed to RRT, or to any other intervention in the past 12 months. Before and after the intervention, the battery of tests described in the next section was administered to participants by clinicians from the Neuropsychiatry Unit where they were recruited.

### 2.3. Measures

*Reading abilities*. Reading was assessed using an Italian battery of standardized tests that provide accuracy and speed scores for reading of passages (‘New MT reading tests for junior high school’: [28]), words and pseudowords (‘Assessment battery for Developmental Reading and Spelling Disorders-2′: [29]).

*Visual attention*. Visual sustained attention was measured using a paper-and-pencil test, the Italian ‘Modified Bell Test’ [30]. In this task, four sheets of paper are placed in front of the participant one after another. Each sheet consists of 35 images of bells (the targets) randomly interspersed with images of other objects (e.g., house, tree, horse, fish). The respondent is instructed to find and mark, by crossing them out, as many of the bells as possible in two minutes. Raw scores for both speed (expressed as the number of correct stimuli marked in the first 30 s spent on a sheet) and accuracy (expressed as total number of correct stimuli marked in the full two minutes) were converted into z-scores and a global score was computed by averaging the raw speed and accuracy scores.

*Auditory attention*. Auditory attention was assessed using the ‘Selective Auditory Attention’ subtest from the Italian ‘Neuropsychological Assessment Battery—BVN’ [31]. In this task, the participant listens to a pre-recorded list of words and is instructed to tap their hand on the table every time they hear a target word (e.g., /sole/). Points are scored for correct responses made within 2 s of stimulus onset. Raw accuracy scores (i.e., number of correct responses) were converted into z-scores.

*Verbal working memory*. Forward and backward digit span subtests from the Italian ‘Neuropsychological Assessment Battery—BVN’ [31] were used to assess verbal working memory. Individual z-scores were computed for each subtest.

*Auditory perception and rhythm reproduction*. Ability to discriminate rhythm and sound was assessed using the ‘Rhythm Task’ from the ‘Assessment Battery for Cross-domain Learning Abilities—Q1 VATA’ [32], a battery of tests designed to measure the main abilities required for school learning (i.e., reading comprehension, meta-comprehension, listening comprehension, writing, study skills, reasoning, numeracy, motor coordination, and rhythm). The ‘Rhythm Task’ comprises two subtests, ‘Duration’ and ‘Rhythm’. In the ‘Duration’ subtest, participants listen to sequences of up to five tones and are instructed to identify the tone which differs from the others in duration. In the ‘Rhythm’ subtest participants listen to sequences of up to 10 beats and are asked to judge whether pairs of sequences are the same or different. Performance in both subtests is expressed as an accuracy z-score. Rhythm reproduction ability was measured using the ‘Rhythm Reproduction Task’ [33], which requires participants to reproduce a set of rhythmic patterns of increasing complexity. Participants are instructed to reproduce the sequences, which are up to eight beats long, by tapping a pencil on the desk; The examiner demonstrates this. Because the only scoring data available for this test are age norms, we scored performance on a 0 to 5 scale: 0 = very low (below the 1st quartile), 1 = low (equal to 1st quartile), 2 = low-medium (between 1st quartile and median), 3 = high-medium (between median and 3rd quartile), 4 = high (equal to 3rd quartile), and 5 = very high (above 3rd quartile).

## 3. Results

### 3.1. Relationships between Baseline Measures

As predicted, baseline measures of reading were strongly correlated (0.58 < r < 0.77). Rhythm discrimination score predicted word reading speed (r = 0.47; *p* < 0.05) and passage reading speed (r = 0.73; *p* < 0.001). Baseline reading abilities were not related to attention, sound length discrimination, rhythm reproduction, or IQ.

### 3.2. Rhythm-Based Intervention Effects

As in the previous studies about RRT efficacy, the intervention produced improvements in word reading speed (F_1,18_ = 16.3; *p* < 0.001; η^2^ = 0.06), nonword reading speed (F_1,18_ = 27.4; *p* < 0.001; η^2^ = 0.12), and passage reading speed (F_1,18_ = 13.8; *p* < 0.005; η^2^ = 0.04). Accuracy (z-score) increased in all tests of reading by an average of 0.37, but this change was not significant. Pre- and post-training z-scores for all reading subprocesses are reported in Table 1.

Turning to the secondary outcomes, the rhythm-based intervention improved rhythm reproduction (F_1,18_ = 10.1; *p* < 0.01; η^2^ = 0.15), visual sustained attention (F_1,18_ = 33.1; *p* < 0.001; η^2^ = 0.31), and auditory selective attention (F_1,18_ = 5.06; *p* < 0.05; η^2^ = 0.09).

### 3.3. Correlations between Initial Neuropsychological Profile and Reading Improvements

The relationships between reading outcomes and specific individual characteristics—such as reading performance prior to intervention, neuropsychological profile, intellectual functioning, and age—were analyzed by calculating correlations. Baseline passage reading speed was negatively correlated with post-RRT passage reading speed (r = −0.59; *p* < 0.01) and nonword reading accuracy at baseline was negatively correlated with improvement in nonword reading accuracy (r = −0.50; *p* < 0.05), as well as with passage reading accuracy at pretest (r = −0.62; *p* < 0.01).

Turning to the other cognitive abilities we assessed, baseline verbal working memory (as measured by the backward digit span task) was negatively correlated with the improvements in nonword reading speed (r = −0.50; *p* < 0.05) and passage reading speed (r = −0.47; *p* > 0.05). Moreover, baseline rhythm discrimination was negatively correlated with post-RRT passage reading speed (r = −0.63; *p* < 0.01). Moreover, visual attention was positively correlated with passage reading accuracy improvement (r = 0.51; *p* < 0.05).

Finally, neither intelligence nor age was correlated with reading improvement during training.

### 3.4. Relationships between Cognitive Abilities Gains and Reading Gains

Improvements in secondary outcomes were correlated with improvements in reading skills. More precisely, passage reading speed improvement was positively correlated with the improvements in verbal working memory (i.e., backward digit span; r = 0.66; *p* < 0.005), rhythm discrimination (r = 0.57; *p* < 0.05), and length discrimination (r = 0.51; *p* < 0.05), but was not correlated with improvements in attention.

### 3.5. Effects Due to the Type of RRT Exercises Used

Improvements in reading were positively correlated with indicators of the amount of RRT participants received (i.e., total number of exercises from each section performed during the training period). Word reading gains were positively correlated with the number of Section 2 (‘Merging’) exercises (r = 0.62; *p* < 0.05) and nonword reading gains were positively correlated with both the number of Section 2 exercises (r = 0.68; *p* < 0.005) and the number of Section 3 (‘Words and Non-words’) exercises (r = 0.57; *p* < 0.05).

## 4. Discussion

Consistent with research identifying rhythm processing as a predictor of reading proficiency [7,23,34,35], we observed a relationship between baseline rhythm discrimination ability and baseline reading speed in our sample. Of the cognitive abilities assessed, only rhythm discrimination was correlated with reading performance; neither attentional abilities nor verbal short-term or working memory were related to reading.

The comparison between pre- and post-training reading performance confirmed that RRT has the potential to improve reading speed, which is consistent with previous research [15,16]. RRT showed a specific impact of reading speed, with a greater effect on reading processes which involve phonological processing and letter-sound mapping (i.e., nonword reading). In contrast with the results of a previous study in which RRT was delivered to junior high school students [16], we found that RRT did not improve reading accuracy. We argue that this inconsistency in results could be due to the high variability in accuracy scores and the age difference between the samples. When we limited our analysis to junior high school students and excluded outliers, a nonparametric Durbin–Conover pairwise comparison indicated an improvement in word reading accuracy (*p* < 0.05), but this result should be interpreted with caution due to the very small size of the subgroup (N = 8). Our design did not include a control condition, because the aim of the study was not to measure RRT efficacy, but to explore how various reading-related cognitive processes contribute to changes in reading ability during the programme.

We checked whether participants’ initial neuropsychological profile accounted for the extent of the improvement that occurred during training. Neither baseline intellectual functioning nor age predicted the impact of the intervention, which implies that all children and preadolescents with dyslexia could benefit from RRT, regardless of their intelligence. Participants with very low passage reading speed at baseline showed the greater improvement in speed during training and reading accuracy results followed a similar pattern: Participants who struggled most to read nonwords or the passage accurately before training showed the greatest improvements in accuracy. These results suggest that the impact of RRT is proportional to the severity of the reading impairment. Improvement in reading speed was negatively correlated with baseline verbal working memory and baseline rhythm discrimination ability, suggesting that children and adolescent individuals with a specific neuropsychological profile—severe impairments in phonological decoding, rhythmic abilities, and verbal working memory—benefit most from RRT. On the other hand, better visual sustained attention was positively associated with improvement in reading accuracy, confirming that RRT is not suitable for participants with attention deficits, due to its visual component.

Visual and auditory attention and rhythmic abilities all improved along with the primary outcome, suggesting that they are involved in the processes underlying the efficacy of RRT. We argue that the visual component of RRT, which is greater when the optional visual cues are used, boosted visual attention, whereas the auditory component boosted rhythmic processing. The large effect of RRT on visual attention could also be interpreted as the result of improved temporal sequencing. As argued by Lallier and colleagues [9], dyslexia-related difficulties in sequential parsing of auditory and visual information during reading lead to deficits in analysis of both the prosodic syllabic contour of words and the visual contour of orthographic units; Thus, improving temporal sequencing should enhance sequential parsing and hence help to focus attention on the signal.

Explorations of the mechanisms involved in RRT showed that improvements in both sound length and rhythm discrimination, and in verbal working memory, were correlated with reading improvements and may, therefore, account for the effect on reading. These cognitive functions appear to be crucial to improvement in passage reading speed, an indicator of reading ability that taps both grapheme-to-phoneme mapping and visual lexical recognition. Evidence that improved auditory perceptual abilities predicted the improvement in reading confirmed the findings by Flaugnacco and colleagues [12], who tested the effect of music lessons on dyslexia. These results can be interpreted within the temporal sampling framework of dyslexia [3], according to which impaired rhythmic sensitivity interferes with development of the phonological skills that are crucial for language and reading acquisition and development. Conversely, attention gains were not correlated with reading improvements; Therefore, they did not account for the primary effect, thus suggesting they did not have a specific role in determining the impact of RRT on reading. An ongoing study measuring the specific effect of the visual cue in RRT on reading is currently testing this hypothesis.

Finally, the type of RRT exercises carried out during training was related to the nature of the improvements in reading: Exercises that targeted word recognition and nonword decoding supported improvements in nonword reading, whereas exercises targeting phonological awareness supported improvements in both word and nonword reading.

As in any small-scale investigation, the small sample size is a possible limitation of the study, but clear-cut effects and associations emerged, suggesting that the results can be considered reliable.

## 5. Conclusions

This investigation explored the cognitive mechanisms underlying the efficacy of a novel, rhythm-based reading intervention that had been found to be effective in improving reading speed in Italian children with dyslexia. Rhythm discrimination ability was found to be strongly related to baseline reading ability and the improvements made during the intervention, which confirms that it plays a crucial role in reading. Basic auditory abilities and verbal working memory were found to contribute to the efficacy of the reading intervention. The pattern of correlations suggests that, to maximize its effectiveness, the RRT intervention should be delivered to children and adolescents who have impairments in reading speed, phonological decoding, verbal working memory, and rhythm discrimination but normal visual attention abilities, as visual attention is needed to cope with the visual component of RRT.

The findings from this study increase our understanding of how RRT works and should be taken into consideration by trainers who wish to use a rhythm-based intervention to improve children’s reading skills.

## Figures and Tables

**Table 1:** Reading variables (z-scores) before and after training.

	Pre-Training Mean (SD)	Post-Training Mean (SD)
**Reading speed**		
Word	−2.56 (0.71)	−2.20 (0.73)
Nonword	−1.92 (0.63)	−1.45 (0.64)
Passage	−2.31 (1.01)	−1.93 (0.83)
**Reading accuracy**		
Word	−3.49 (3.29)	−3.07 (3.48)
Nonword	−2.37 (2.42)	−1.90 (2.37)
Passage	−1.54 (1.87)	1.31 (1.83)
